# Training Ghanaian frontline healthcare workers in public health surveillance and disease outbreak investigation and response

**DOI:** 10.11604/pamj.supp.2016.25.1.6179

**Published:** 2016-10-01

**Authors:** Donne Kofi Ameme, Kofi Mensah Nyarko, Edwin Andrews Afari, Simon Antara, Samuel Oko Sackey, Fred Wurapa

**Affiliations:** 1 Ghana Field Epidemiology and Laboratory Training Programme (GFELTP), School of Public Health, University of Ghana, Accra, Ghana; 2 Disease Control and Prevention Department, Ghana Health Service, Accra, Ghana; 3 Public Health Solutions, Ghana

**Keywords:** Training Ghanaian, frontline health workers, Ghana

## Abstract

**Introduction:**

Beyond initial formal academic education, the need for continuous professional development through in-service workforce capacity improvement programs that are aimed at enhancing knowledge and skills of public healthcare workers has assumed immense priority worldwide. This has been heightened by the on-going Ebola Virus Disease outbreak, which is exposing the weak public health systems in West Africa. In response to this need, the Ghana Field Epidemiology and Laboratory Program organized a short-course for frontline health workers in the Greater Accra region of Ghana in order to augment their surveillance and outbreak response capacity.

**Methods:**

Human and veterinary health workers were trained using Field Epidemiology and Laboratory Training Program short course model. A two-week didactic course was conducted with a 10-week field placement. Evaluation of the course was done by assessment of participants’ outputs during the training as well as pretest and posttest methods.

**Results:**

A total of 32 frontline health workers from both the human and veterinary health services benefited from the two-week initial training of the 12-week course. There was a significant gain in knowledge by the participants after the training course. Participants developed concept papers and implemented their fieldwork projects. Overall assessment of the workshop by the participants was very good.

**Conclusion:**

Capacity of the health workers has been improved in the area of public health surveillance, outbreak investigation and response. We recommend a scale-up of this training course to other regions.

## Introduction

Strengthening public health systems has become a recognized priority globally [1] particularly in Africa where public health systems have been described as weak [2]. The Ebola Virus Disease epidemic of unprecedented proportion which is unfolding in parts of West Africa has exposed this weakness the more. A major area of weakness is the unavailability of skilled and capable health workforce at all levels of the health care system [2]. Availability of a competently trained workforce is a prerequisite for effective functioning of health systems [1, 3]. Positive linkage between health workforce strength and health outcome have been demonstrated [2, 4, 5]. Guinea, Sierra Leone and Liberia, the three countries most affected with the on-going EVD outbreak, lack specific programs to adequately train and support a public health workforce that is capable of operating an effective surveillance and response system [2]. The repercussions of this are evidenced by the manner in which public health infrastructures of these countries have been overwhelmed by the EVD outbreak. The contrary can be said of Nigeria which invests in the training and development of skilled multidisciplinary health care professionals in surveillance and outbreak investigation through the Field Epidemiology Training Program [6] who were swiftly activated and supported to control the EVD outbreak. Indeed the economic implications of the raging EVD epidemic outweighs the investment in developing and supporting a competently trained workforce capable of responding to public health challenges [2]. Immediate actions by countries geared towards developing the capacity of local public health workforce and supporting them to effectively operate a multi-disease public health surveillance and response systems has been highly recommended [2]. An important and well-recognized tool for achieving this feat is the 2-year full-time, competency-based training program in applied epidemiology being run by Field Epidemiology Training Programs (FETPs) globally [1, 7, 8]. These programs patterned after CDC’s Epidemic Intelligence Service (EIS) program [7], are run as joint training courses for both veterinary and the medical professionals (nurses, laboratory scientists and medical doctors) based on the “one health” concept [9, 10]. These trainings produce highly skilled staff for high-level leadership roles in the various Ministries of Health and allied agencies [11]. The limitation is the lack of skilled professionals at the lower level or the primary healthcare level who are usually the first port of call for most public health emergencies. This imposes challenges to their capacity to respond adequately to address public health challenges. The three-tiered curriculum training approach, which addresses the capacity at all the various levels of care, fills this gap. The first tier (advanced training) is the usual 2-year training of high-level field epidemiologists. The second tier is the intermediate level, which is a 9-month training program and the third tier, which is the basic 3-month training curriculum, applies to workers at the lowest level. Developing sub-national capacity by training a core team of frontline medical and non-medical personnel in field epidemiology and supporting them in multi-disease public health surveillance and response therefore is essential. Since the 2-year FETPs are designed to train a select few [12] probably as a result of resource limitations, graduates of these programs are inadequate to meet the needs of the population. Supplementing with trained lower level frontline healthcare workers through FETP driven competency-based short courses [12–14] becomes a necessary option. In Ghana, the University of Ghana and the Ghana Health Service has collaborated in designing and conducting competency-based short courses for district level health staff [15] to address this need. The Ghana Field Epidemiology and Laboratory Program (GFELTP) which has also been developing local epidemiological capacity [9, 15] has taken this a step further by collaborating with stakeholders other than the Ghana Health Service such as the Veterinary Services Division to roll out a nation-wide short course training program for frontline health workers in order to respond to their capacity development need. This training program has become necessary in the face of the lack of capacity of the frontline health workers to respond adequately to the many public health emergencies that occur in the country. The ongoing cholera outbreak in Ghana and the Ebola scare following the Ebola Virus Disease outbreak in West Africa makes this training very timely. The first in the series of these training programmes was held in the Greater Accra Region of Ghana with the objective of strengthening capacity of frontline healthcare workers in the region in the area of public health surveillance and disease outbreak investigation and response. This paper seeks to provide a comprehensive account of the programme design, implementation, output and outcome as well as showcase effective capacity strengthening interventions.

## Methods

### Program Design and Implementation

The course was designed as a joint training of both veterinary and medical professionals based on the “one health” approach. The curriculum includes both didactic and fieldwork components akin to the FETP. The didactic component of the course was on a full time residential basis. The fieldwork was preceded by relevant presentations and case studies to set the tone for a more detailed fieldwork of 10 weeks duration under supervision. The 2-week competency based training workshop was organized from the 24th November to 5th December 2014. The training was on public health surveillance and disease outbreak investigation and response with a focus on Ebola Virus Disease. The training was modeled after the FELTP short courses for public health workers. The participants were assisted to select their proposed projects in the following three areas: surveillance system evaluation, outbreak investigation and response, data analysis for public health action. A dissemination workshop was held at the end of the project where participants were given the opportunity to make oral presentations of their projects for input. The GFELTP organized the workshop in collaboration with the Ghana Health Service and the Veterinary Services Division of the Ministry of Agriculture.

### Pre-workshop tasks

Two weeks prior to the workshop, the curriculum of the course was developed and the facilitators met twice to review the curriculum to meet the standards of the participants. The various topics to be covered were selected at these meetings. The taught courses ranged from basic statistics and analysis of surveillance data, public health surveillance, outbreak investigation and response, and use of Microsoft Excel and Epi Info software for basic analysis. The authorities of the Ghana Health Service and the Veterinary Services Division were engaged to identify and enroll members of their staff who were most likely to benefit from the training.

### Location

The training workshop took place in, Accra, Ghana.

### Facilitators

The course was facilitated by faculty and alumni of the GFELTP as well as a public health consultant of the Ghana Public Health Solutions. The facilitators have immense experience in the area of field epidemiology and are well versed in knowledge transfer skills.

### Course materials

The participants were provided with the relevant reading materials and online sources of information. The materials were adapted from the FETP curriculum and tailored to the needs of the participants.

### Evaluation

Evaluation of the training was done through pretest posttest, feedback from participants during and after the training, and through the quality of written and oral training-related assignments. Participants were given the opportunity to assess the workshop in terms of quality and whether it met their expectations. The participants responded anonymously to a set of questions to assess the overall quality and the content of the course. They also gave their impressions and made some recommendations for improvement. The participants’ assessment of various aspects of the training workshop was done by using a five point Likert-type scale, whose responses ranged from “poor” to “excellent”. The participants’ knowledge gain was assessed by a pretest posttest interventional study design [16]. Assessment of their baseline knowledge was done by set of 16 questions covering all the major areas of the course content. The total knowledge score ranged from 0-25. The level of knowledge was classified into three categories: poor (below 11), fair (between 11 and 15) and good (16 or more). After the two week training, assessment of the participants gain in knowledge was done using the same set of questions as a posttest. Participants used the same unique identification codes for both the pretest and posttest examinations. The unique identification codes were known only to the participants. Data entry, cleaning and analysis was done using Microsoft Excel software.

## Results

A total of 32 frontline junior and mid-level healthcare workers comprising disease control officers, information officers, public health nurses and medical officers from the Ghana Health Service as well as field technicians from the Veterinary Services Department of the Ministry of Agriculture attended the workshop. Majority, 21(65.6%) of the participants were males with 22 (68.8%) of them having more than 5 years work experience (Table 1).

**Table 1:** Characteristics of training participants, Accra, Ghana-2014

Characteristics	Frequency	Percentage
**Sex**		
Males	21	63.64
Females	11	33.33
**Duration in service**		
0-5 years	10	30.30
6-10 years	11	33.33
>10 years	11	33.33
**Cadre**		
Medical Officer	6	18.18
Disease Control Officer	11	33.33
Nurse	5	15.15
Veterinary Field Technician	5	15.15
Health Information Officer	6	18.18

The response rate for the pretest and posttest was 93.94%. Analysis of the evaluation forms filled by the participants revealed that the participants were impressed with the quality and content of the workshop. Six out of the 31 participants, who responded, rated the overall quality of the workshop as excellent and 13 rated it as very good (Table 2).

**Table 2:** Participants’ assessment of the training course, Accra, 2014

Output Measure	Response				
	Poor	Fair	Good	Very Good	Excellent
	n (%)	n (%)	n (%)	n (%)	n (%)
Quality of lectures	0 (0.00)	0 (0.00)	0 (0.00)	14 (45.16)	17 (54.84)
Hands on session	0 (0.00)	0 (0.00)	0 (0.00)	18 (58.06)	13 (41.94)
Reading materials	0 (0.00)	0 (0.00)	7 (22.58)	18 (58.06)	6 (19.35)
Pace of course	0 (0.00)	0 (0.00)	6 (19.35)	19 (61.29)	5 (16.13)
Overall quality of course	0 (0.00)	1 (3.23)	10 (32.26)	13 (41.94)	6 (19.35)

They however recommended among other things that the duration of the residential component of the training be extended to at least four weeks. Thirty (96.77%) of the respondents expressed confidence in responding to local health events and priorities. Before the training workshop, 9 (27.27%) participants had poor level of knowledge, 22 (66.67) had fair level of knowledge and 2 (6.06%) had good level of knowledge on public health surveillance and outbreak investigation and response. After the training, none of the participants had poor knowledge, 3 (9.09%) had fair knowledge and the rest 30 (90.90%) had good knowledge. The mean scores (+/- standard deviation) were 12.00 (+/- 2.70) and 18.39(+/- 2.49) for the pretest and posttest respectively. The difference of 6.39 was statistically significant at 5% confidence level. (t(31)=13.37, p< 0.001). Multiple linear regression using participants’ knowledge score after the training as the dependent variable and the covariates of sex of participants, knowledge score before the training, duration of service in health sector and duration of service in the field of public health showed only the length of service was significantly predictive of the participants knowledge score after the training (t0.05 (28)= 3.22). Sex of the participants (t0.05 (28)= 1.94), knowledge score before the training (t0.05 (28)= 2.03) and duration of service in field of public health (t0.05 (28)=0.56) were not useful in estimating the knowledge score after the training. About 27% percent of variability on the participants’ knowledge scores after the training was attributed to the participants’ knowledge score before the training, the participants’ length of stay in the health sector and the duration of public health service, at statistically significant levels (adjusted r2=26.6, p=0.01). The overall probability of gaining knowledge after the training course was 100%.

### Output

A total of 32 frontline health workers from the veterinary and human health sectors in the Greater Accra Region were trained on basic concepts in field epidemiology and supported for a fieldwork. The participants identified projects they would like to carry out during their fieldwork. A total of thirty-two concept papers were produced on the various proposed fieldwork projects. All participants made oral presentations of their proposed projects for technical input from the facilitators. This was done to make the proposed projects technically sound and provide a framework for successful fieldwork.

### Outcome

The outcome measure was the gain in knowledge of the participants and to what extent this knowledge gain has been put to appropriate use. The gain in knowledge was evident in the improvement in the mean knowledge score of the participants post training. By the end of the course, the participants were equipped with the skills to conduct public health surveillance and outbreak investigation tasks with minimal supervision. The translation of the knowledge to practice was assessed by the fieldwork and mentorship component during their field placement and in the feedback workshop. Participants have used the knowledge gained to analyze surveillance data and develop threshold for diseases in their respective districts. Some participated actively in responding to major outbreaks and others evaluated public health surveillance systems. Key outputs produced by the participants at the dissemination conference included 32 written reports of the respective projects. A total of 27 oral and five poster presentations on surveillance data analysis for action, surveillance system evaluation and outbreak investigation were made to stakeholders at the dissemination conference.

## Discussion

The training of this well integrated mass of health workers is a critical step in responding to the much-recognized unmet health workforce need plaguing African nations [17]. The training of the health workers on this important aspect of public health has yielded significant gain in knowledge. The mean scores of participants after the training was higher than the mean score before the training indicative of an improvement in the knowledge of the participants in public health surveillance and outbreak investigation and response. All the participants had gained some knowledge after the training suggesting a potential for translation into practice. The confidence expressed by the participants in their capacity to respond to outbreaks gives indication of a prepared public health workforce. These participants will serve as important resources in their various districts to be deployed in public health emergencies, a need that has come to the fore in the light of the devastating effect of Ebola Virus Disease in West Africa. The capacity built will help the districts in responding effectively to major public health problems in the region. Training of health professionals in field epidemiology in Ghana and elsewhere, though at a higher level, has yielded similar valuable results [6, 9, 14]. The significant correlation between length of service and knowledge score after the training was not surprising as the content of the training course was competency based and service delivery oriented. Correlation between knowledge and length of service has been found elsewhere [18]. The training therefore served as a refresher for participants with long duration of service as they had been practising most of the skills prior to the three-month training.

## Conclusion

A good mix of animal and human health professionals has been trained in public health surveillance and disease outbreak investigation and response. The surveillance and outbreak response capacity of frontline veterinary and human health professionals in the Greater Accra region of Ghana has been strengthened. There was a significant gain in knowledge after the training session indicative of the potential for effective public health action. The scale-up of this training course to include other regions will be beneficial.
